# Variations in product in reactions of naphthoquinone with primary amines

**DOI:** 10.1186/1860-5397-3-10

**Published:** 2007-03-01

**Authors:** Marjit W Singh, Anirban Karmakar, Nilotpal Barooah, Jubaraj B Baruah

**Affiliations:** 1 Department of Chemistry, Indian Institute of Technology Guwahati, Guwahati 781 039 Assam, India

## Abstract

Reaction of 1,2-naphthoquinone with primary amines gives a 2-amino-1,4-naphthoquinone derivative which is equivalent to 1,2 to 1,4 carbonyl transposition. For example the reaction of 1,2-naphthoquinone with 4-methoxyaniline gives 2-(4-methoxyanilino)-naphthoquinone-1,4-(4-methoxyanil) (**1**) and with *n*-butylamine gives 2-(butylamino)-naphthoquinone-1,4-butylimine (**2**) respectively. The compounds **1** and **2** are characterized by X-ray crystallography; they have hydrogen-bonded dimeric structures. Similar reaction of 1,4-naphthoquinone with 3-picolylamine and 4-picolylamine gives the corresponding 2-amino 1,4-naphthoquinones; two products are characterized by X-ray crystallography. The reaction of 1,4-naphthoquinone with 4-aminothiophenol and 1,4-naphthoquinone with 4-aminophenol are compared. The former leads to C-S and the latter to C-N bond formation. The reaction of 1,4-naphthoquinone with 4-aminothiophenol in an NMR tube is studied to explain that 2-(4-anilinothiolato) 1,4-naphthoquinone derivative to be the sole product in the reaction.

## Background

Amino quinones are used as medicines, [1–3] herbicides [4] and they also show interesting redox switching properties. [5] Amino quinones are formed in the reactions of different amines with quinones. [6–13] For example 1,4-benzoquinone reacts with primary amines to give 2,5-diamino 1,4-benzoquinones; similar reaction of 1,4-naphthoquinone with primary amines results in the formation of 2-amino 1,4-naphthoquinones. [13] However, the product formed from such simple reaction of amine with various quinones has much scope for exploration, especially in terms of synthesis of electro/photoactive supramolecular assemblies or polymers. In this communication we describe preliminary account of results obtained on the equivalence of reaction of 1,2-naphthoquinone and 1,4-naphthoquinone with primary amines.

## Results and discussion

**Scheme 1:**
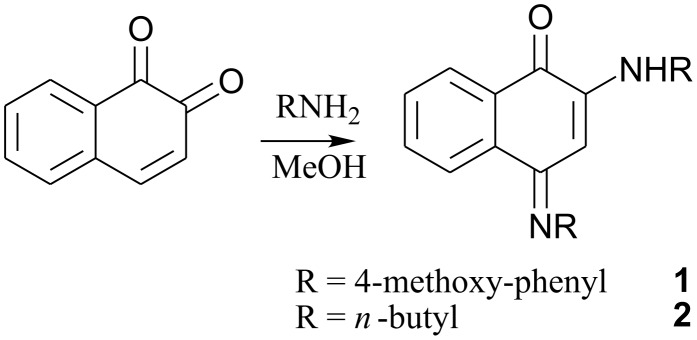
Reaction of 1,2-naphthoquinone with primary amines.

It is observed that the reaction of 1,2-naphthoquinone with primary amines leads to incorporation of imino group at 4-position as illustrated in Scheme 1. During such reaction an amine functional group replaces the carbonyl group present at the 2-position of 1,2-naphthoquinone. The products thus formed have the structural features of 1,4-naphthoquinone. Illustrative examples of the reactions of amines, namely 4-methoxyaniline and *n*-butylamine, with 1,2-naphthoquinone are shown in Scheme 1. The compounds **1** and **2** are characterized by determining their crystal structures and also by other spectroscopic techniques. The formation of products **1** and **2** is interesting as in the case of 1,4-benzoquinone and 1,4-naphthoquinone we did not observe condensation reaction between either of the carbonyl group with amines under ambient conditions (for experimental please see Supporting Information File 1).

It was earlier reported in the literature that the reaction of *N*-phenyliminophosphorane with 1,2-naphthoquinone results in the formation of 2-anilino-naphthoquinone-1,4-anil [14] and in our present investigation we have observed that such products can be prepared just by reaction of 1,2-naphthoquinone with a primary amine. Further to this there has been report in the literature in which the 1,2-naphthoquinone having sulfonate group at *4*-position were used to prepare 1,4-naphthoquinone derivatives. [15] Thus, our method is mild and is advantageous in terms of synthetic procedure.

**Figure 1:**
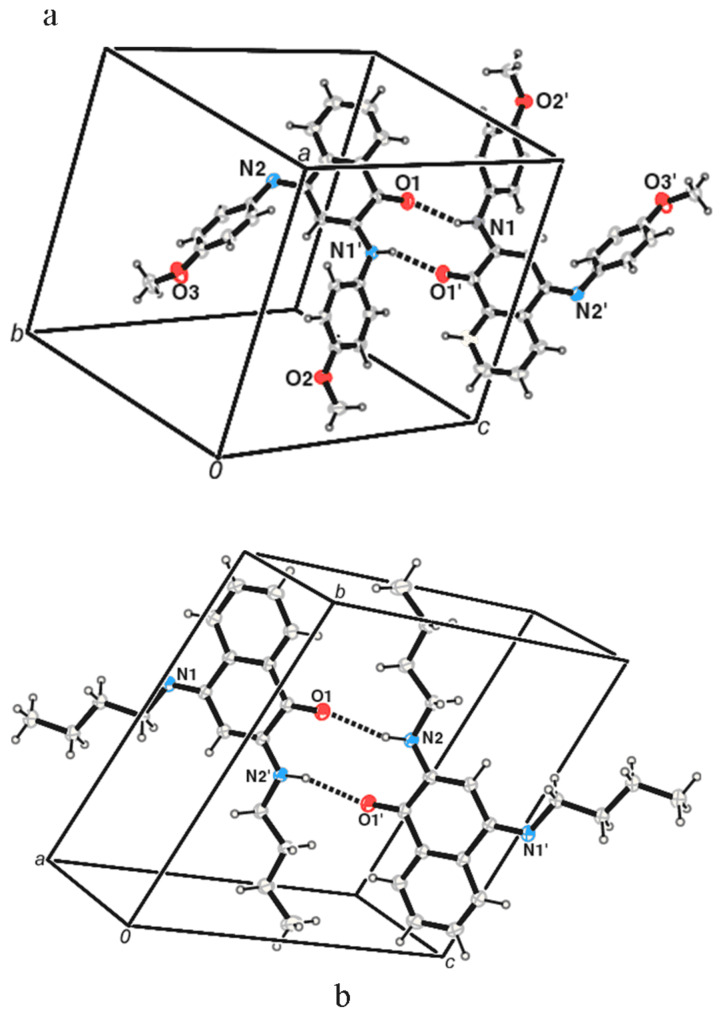
The solid state structure of (a) **1** and (b) **2** (drawn with 20% thermal ellipsoids).

The solid-state structures of the compounds **1** and **2** are shown in the Figure 1 (for numbering of atoms in X-ray structure please refer to supplementary CIF files). The compound **1** has the carbon oxygen double bonds distance C1-O1 as 1.220 Å. In the molecule **1** there are two carbon nitrogen bonds one of which corresponds to a single bond (C2-N1 1.359 Å), and the other to a double bond (C4-N2 1.297 Å). In the crystal lattice, the compound **1** forms dimeric assembly held by hydrogen bonding interaction via N-H. . . O (d_D-H_, 0.825 Å, d_H-A_, 2.210 Å, d_D..A_, 2.938 Å, <D-H. . . A, 147.40°). The compound **2** has also similar features as that of the **1** and it has C=O distance C4-O1 as 1.231Å. It also has two different carbon-nitrogen bond distances N2-C3 as 1.353 Å, N1-C1 as 1.286Å corresponding to a C-N and a C=N respectively. In the solid state the molecules of **2** are held by hydrogen bonding interactions between N-H. . . O leading to dimeric structures with d_D-H_, 0.889 Å, d_H-A_ 2.287 Å, d_D-A_ 3.151 Å, <D-H. . . A 163.98° respectively.

Formation of the imine derivative at the 4-position in these reactions may be attributed to the concomitant formation of 4-amino derivative along with the imine at 2-position. Tautomerism of the imine and amine group leads to the desired product. Valence tautomerism is commonly observed in metal complexes of 1,2-quinonic compounds. [16–22] In our reactions two moles of amines are used; with one mole of 1,2-naphthoquinone the same product is obtained as with 1:1 stoichiometry between amine and 1,2-naphthoquinone. It may be noted that similar products from the reaction of 1,2-naphthoquinone with secondary amines such as diethylamine were not formed. There are two possible paths for formation of the product illustrated in Scheme 1: that amine can first attack the carbon at 4-position of the ring followed by formation of a 4-amino, 1–2-naphthoquinone derivative, which is followed by imine formation at 2-position. The second alternative is the formation of imine followed by attack of the amine at 4-position to give the desired product. We have not been able to isolate any intermediate but based on the established fact that the presence of a carbonyl group favors γ-attack on a α-β unsaturated carbonyl over an imine; we would like to put forward the first path. But it is still not clear about the aromatization process, whether it is a unimolecular, or a bimolecular process. Work is in progress to establish this. However, at the moment we have evidence that in the reaction between 4-amino-phenol and 1,4-benzoquinone, 1,4-dihydroxybenzene is formed as side product. This suggests involvement of 1,4-benzoquinone in aromatisation by abstracting hydrogen during product formation. In view of absence of values for redox potentials of the intermediates, we prefer to limit our discussion on this issue. Lack of solubility of the products makes mechanistic study difficult. We have also studied the reaction of 1,4-naphthoquinone with di-isopropyl amine and found that a highly insoluble product is formed, due to purification problem as well as solubility it remains uncharacterized.

**Scheme 2:**
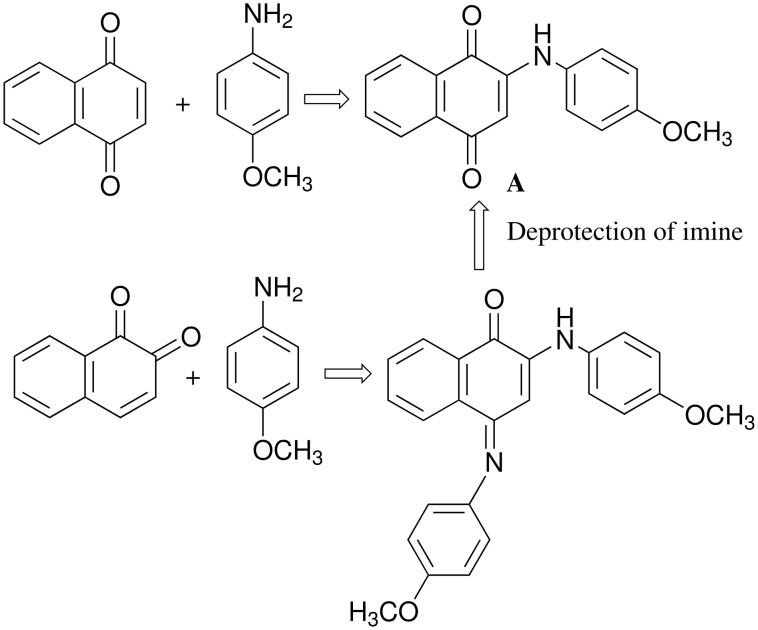
Equivalence of reactivity between 1,2 and 1,4-naphthoquinone.

**Scheme 3:**
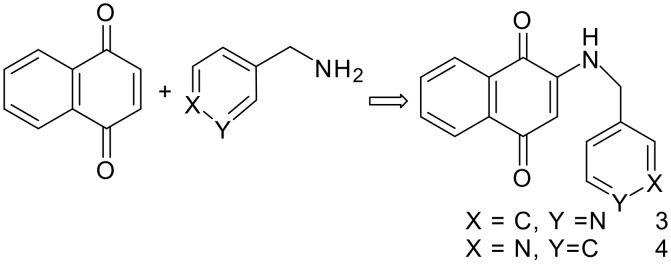
The reaction of picolylamine with 1,4-naphthoquinone.

In a sense, both the reactions of 1,2-naphthoquinone and 1,4-naphthoquinone with primary amines are equivalent, as both reactions lead to formation of 2-amino 1,4-naphthoquinone derivatives as illustrated in Scheme 2. We have already reported the reaction of *p*-methoxy aniline with 1,4-naphthoquinone leading to the 2-(*p*-methoxy-anilino) 1,4-naphthoquinone along with its crystal structure. [13] We extended our study to incorporate a pyridine-containing tether with a view to make a new series of compounds that may have anion binding ability on protonation or have complex forming ability with metals. The incorporation of pyridine ring to a quinone has also another facet as we have recently observed that in picolyl derivative of 1,8-naphthalimides the π-π interactions are governed by the position of the nitrogen in the pyridine ring. [23] Thus, we prepared two picolyl derivatives of 1,4-naphthoquinone from independent reactions of 3-picolylamine and 4-picolylamine with 1,4-naphthoquinone. These reactions resulted in the corresponding 2-amino substituted 1,4-naphthoquinones as illustrated in Scheme 3.

**Figure 2:**
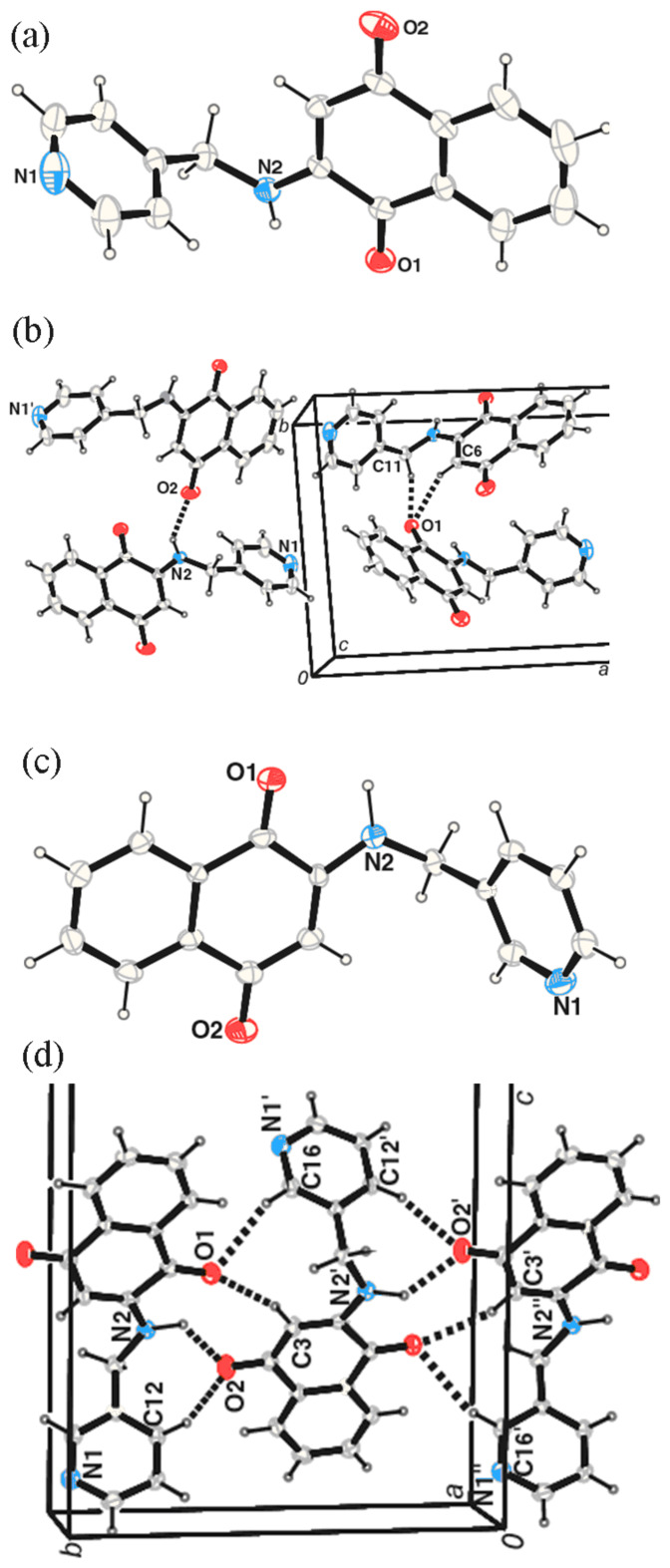
(a) The crystal structure of **3** and (b) weak interactions in **3** leading to self-assembly, (c) Structure of **4** (drawn with 20% thermal ellipsoid) (d) the N-H. . . O interactions C-H. . . O interactions in **4** (a part of the unit cell is shown).

The products from these reactions are characterized by different spectroscopic techniques and also by X-ray crystallography. The crystal structures of **3** and **4** are shown in Figure 2. In solid state the weak interactions among the molecules lead to formation of self-assemblies of **3** and **4**. Among the weak interactions N-H. . . O, C-H. . . O and C-H. . . π interactions play roles in the self-assembly formation as illustrated in Figure 2.

Some of the important weak bonds responsible for hydrogen bonding assemblies in **3** C6-H. . . O1 (3.303Å, 153.07°), C11-H. . . O1 (3.540Å, 143.41°), N2-H. . . O2 (2.877Å, 140.18°) interactions and these interactions are shown in are C16-H. . . O1 (3.486Å, 159.57°), C3-H. . . O1 (3.472Å, 162.51°), N2-H. . . O2 (3.005Å, 126.80°), C13-H. . . O2 (3.353Å, 143.73°) (refer Figure 2b). In addition to this the C12 and C13 carbon centers are involved in C-H. . . π interaction with C11-H bond of another neighboring molecule. It is the case with compound **4** which forms self-assembly through Figure 2d. In addition to these there is C-H-π interaction through C13-H. . . C5 (3.389Å) and C11-H. . . π (3.814 Å, H. . . A 2.865Å, 166.14°) interaction (for crystallographic numbering please see CIF files in Supporting Information File 2).

**Scheme 4:**
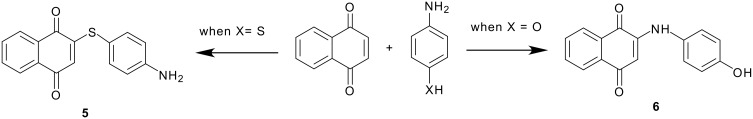
The reaction of 1,4-naphthoquinone with 4-aminothiophenol and 4-aminophenol.

**Figure 3:**
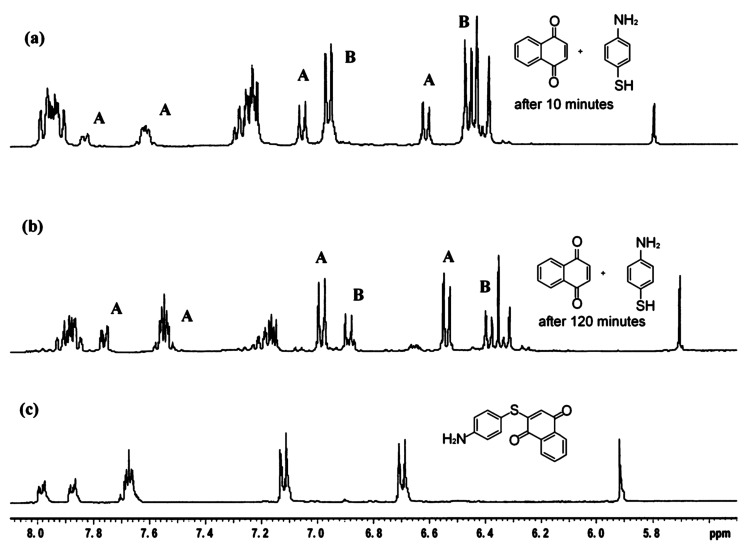
The ^1^HNMR spectra (400 MHz) of the reaction mixture of 1,4-naphthoquinone with 4-amino thiophenol (1:1 molar ratio in CD_3_OD) (a) after 10 minutes, (b) after 2 hrs (c) the purified product from the reaction.

**Figure 4:**
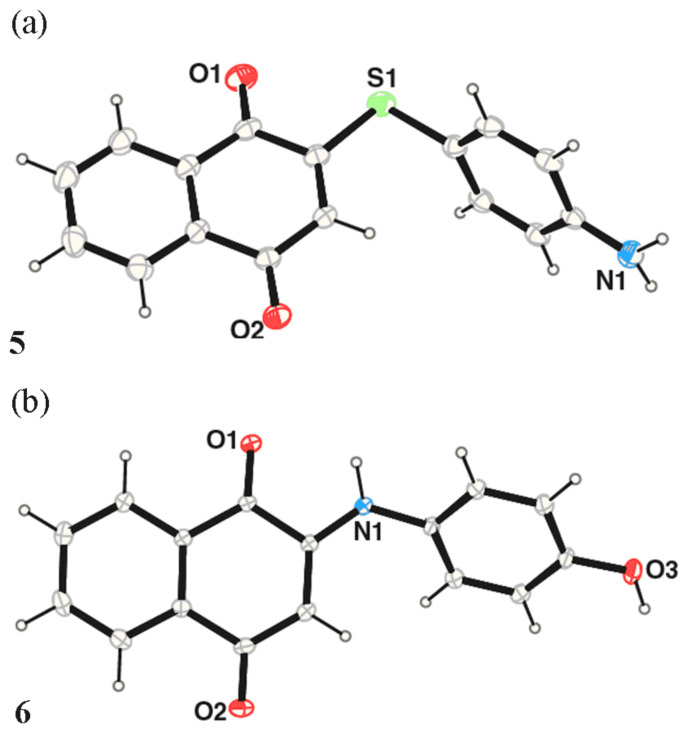
The structure of the products from the reaction of 1,4-naphthoquinone with (a) 4-aminothiophenol (b) 4-aminophenol (20% thermal ellipsoid).

In order to elucidate the reaction mechanism we have studied the reaction between 1,4-naphthoquinone with 4-amino thiophenol (1:1 molar ratio) in deuterated methanol by reacting them in an NMR tube. The reaction products are compared with the purified isolated product (Figure 3c). Surprisingly in this reaction the product formed is through C-S bond formation rather than C N-bond formation (Scheme 4). The presence of a few well-separated peaks of the products from the starting materials in the NMR spectra (designated by A) shows the formation of the one product with time. As time progresses the two doublets from 4-aminothiophenol (designated by B in Figure 3) decreases in intensity. In each case the proton chemical shifts are compared with the residual undeuterated methyl-peak in the deuterated methanol. It is observed that NMR spectra of the reaction mixture comprise of only the signals from the product and the starting materials, however, the peak positions varied with time. The product 2-(4-thiolanilino) 1,4-naphthoquinone was purified at the end of the reaction and confirmed by recording its ^1^H NMR (Figure 3c), IR and elemental analysis. We do not have complete understanding of the mechanism but the observations hint towards a radical mechanism rather than an ionic mechanism. In contrast to the reaction of 4-aminothiophenol with 1,4-naphthoquinone the reaction of 4-aminophenol gave the expected product through C-N bond formation. The structures of the compounds are further determined by X-ray crystallography and are shown in Figure 4. The compound **5** also self-assemble in solid state and remain as dimer; which further form hydrogen bonded chains. The compound **6** forms self-assembly through N-H. . . O interactions and have chain structure. The two aromatic rings are perpendicular to each other. The C-S bond formation reaction can be extended to other thiols such as thiophenol and 4-methoxythiophenol, 4-bromothiophenol etc. with 1,4-naphthoquinone as well as 1,4-benzoquinone. The difference in the case of reaction of 1,4-benzoquinone with primary amine from that of reaction of 1,4-benzoquinone with thiophenol is that the former gives disubstituted product [13] whilst the latter leads to mono-substituted product.

In conclusion the reaction of 1,2-naphthoquinone as well as 1,4-naphthoquinone with primary amines are compared. The amino-substituted derivatives formed from both systems are amino-substituted at the 2-position, which in the case of 1,2-naphthoquinone is similar to incorporation of a carbonyl group at the 4-position by 1,2 to 1,4 transposition of carbonyl group. The reaction of 2-aminophenol with naphthoquinone leads to C-N bond formation whereas similar reaction of 1,4-napthoquinone leads to C-S bond formation. The advantage of these reactions is mildness and versatility. C-N as well as C-S bond formation can be achieved in quinonic compounds under mild conditions. However, limited mechanistic understanding of the reactions is the identification of a suitable system for obtaining homogeneous solutions with primary amines.

## Supporting Information

File 1 Supporting information. Experimental procedure and spectral data of products and the crystallographic information.

File 2 CIF. Crystallographic information files for compound **1–6**.
