# Supplementary material for: Variations in product in reactions of naphthoquinone with primary amines
Source: Beilstein J Org Chem. 2007 Mar 1;3:10. doi: 10.1186/1860-5397-3-10 (PMC1838918; doi:10.1186/1860-5397-3-10)
Supplement: File 1 — Supporting information. Experimental procedure and spectral data of products and the crystallographic information. [file Beilstein_J_Org_Chem-03-10-s001.doc]

**Variations in product in reactions of naphthoquinone with primary amines**

W. Marjit Singh, A. Karmakar, Nilotpal Barooah, Jubaraj B. Baruah*

Department of Chemistry, Indian Institute of Technology Guwahati, Guwahati 781 039 Assam, India

**Experimental:**

***General procedure for synthesis of compounds 1-2:*** 1,2-naphthoquinone (1mmol) and corresponding amine (2mmol) under consideration were mixed together in methanol (10ml) to obtain homogeneous solutions. The solutions were stirred at room temperature for 3days in open air and the residue obtained was filtered. The residues were recrystallised to obtain the desired product.

In a similar manner compound **3**,**4**,**5** and **6** were prepared from reaction of 1,4-naphthoquinone with corresponding amine in 1:1 molar ratio.

**Spectroscopic data**

**1**: Isolated yield 70%. ^1^HNMR (DMSO-d_6_): 8.54 (d, J=4Hz, 1H), 8.18 (dd, J=4, 4Hz, 1H), 7.75-7.72 (m, 2H), 7.21-7.17(m, 1H), 6.96-6.87 (m, 2H), 6.71(s, 2H), 5.95 (s, 1H), 3.82 (s, 6H). IR (KBr, cm^-1^): 3323 (br), 1654(s), 1604(s), 1516(s), 1338(m), 1292(m), 1236(s), 1182(m), 1029(s), 837(s), 775(m), 1097(w), 715(m). UV-vis (λ_max_, Methanol) 486nm (ε = 0.43 X10^3^ mol^-1^ cm^-1^).

**2**: Isolated yield 68%. ^1^HNMR (CDCl_3_): 8.42 (d, J=4Hz, 1H), 8.10 (d, J=4Hz, 1H), 7.61(t, J=4Hz, 1H), 7.48 (t, J=4Hz 1H), 5.97 (s, 1H), 5.45 (bs, 2H), 3.74 (t, J=4Hz, 2H), 3.13 (q, J = 4Hz, 2H), 1.82 (pentate J =8Hz, 2H), 1.69 (pentate J=8Hz , 2H), 1.51 (m, 4H), 1.01(q, J= 8Hz, 6H). IR (KBr, cm^-1^) 3436 (s), 3342(s), 2954(s), 2928(s), 2860(s), 1654(s), 1597(s), 1576(m), 1510(m), 1476(w), 1331(s), 1097(w), 765(m), 735(s). UV-vis (λ_max_, Methanol) 450nm ( ε = 0.27 X10^3^ mol^-1^ cm^-1^)

**3**: Isolated yield 84%. ^1^HNMR (CDCl_3_) 8.58(d, J=8Hz, 2H), 8.06(d, J=8Hz, 2H), 7.72 (t, J=8Hz , 1H), 7.65 (t, J =8Hz, 1H), 7.24 (s, 1H), 7.20 (d, J=8Hz, 1H), 6.26 (s, 1H), 5.65 ( s, 1H), 4.40(s, 2H). IR(KBr, cm^-1^) 3431 (s), 3332(s), 1677(s), 1602(s), 1594(s), 1568(s), 1559(s), 1504(s), 1358(m), 1340(m), 1259(s), 1126(m), 733(m). UV-vis (λ_max_, Methanol) 436nm ( ε = 0.5X10^3^ mol^-1^ cm^-1^)

**4**: Isolated yield 72%. ^1^HNMR (CDCl_3_): 8.58 (m, 2H), 8.06 (t, J= 8Hz, 2H), 7.72 (t, J=8Hz, 1H), 7.63-7.59 (m, 2H), 7.31-7.28 (m, 1H), 6.22 (s, 1H), 5.78 (s, 1H), 4.40 (m, 2H). IR(KBr, cm^-1^) 3430 (s), 3349(s), 3312(s), 1675(s), 1610(s), 1601(s), 1568(s), 1500(s), 1358(s), 1339(m), 1264(m), 1123(s), 748(m), 729(s), 711(s). UV-vis (λ_max_, Methanol) 468nm (ε = 0.5X10^3^ mol^-1^ cm^-1^).

**5**: Isolated yield 90%. ^1^HNMR (CDCl_3_): 8.12(d, J=8.8Hz, 1H), 8.01(d, J=8.8Hz, 1H), 7.71(m, 2H), 7.17(d, J=8.8Hz, 2H), 6.75(d, J=8.4Hz, 2H), 5.91(s, 1H), 3.98(bs, 2H).

IR(KBr, cm^-1^): 3458(s), 3364(s), 1661(s), 1634(s), 1591(s), 1556(m), 1499(w), 1334(w), 1319(w), 1301(s), 1249(s), 1217(w), 1119(w), 1065(m), 852(w), 781(w), 704(m), 531(w).

**6**: Isolated yield 68%. ^1^HNMR (DMSO-d_6_): 9.66(s, 1H), 9.07 (s, 1H), 8.02 (d, J = 8Hz, 1H), 7.92 (d, J=7.2Hz, 1H), 7.83 (t, J= 7.2Hz 1H), 7.76 (t, J= 7.2Hz , 1H), 7.14(d, J=8Hz 2H), 6.82 (d, J=8Hz, 2H) 5.86 (s, 1H). ^13^CNMR (DMSO-d_6_): 182.3, 181.8, 155.4, 147.2, 135.0, 132.5, 130.5, 129.0, 126.1, 125.3, 115.9, 100.8. IR(KBr, cm^-1^): 3304(s), 1670(m), 1624(s), 1601(s), 1531(m), 1519(m), 1438(m), 1365(s), 1332(w), 1268(s), 1161(w), 1124(w), 991(w), 821(w), 775(w), 724(m), 672(w).

**X-ray crystallography:**

X-ray crystallographic data were collected at 296K with Mo*_Kα_* radiation (λ = 0.71073 Å) using a Bruker Nonius SMART CCD diffractometer equipped with graphite monochromator. The SMART software was used for data collection and also for indexing the reflections and determining the unit cell parameters; the collected data were integrated using SAINT software. The structures were solved by direct methods and refined by full-matrix least-squares calculations using SHELXTL software. All the non-H atoms were refined in the anisotropic approximation against *F^2^* of all reflections. The H-atoms, except those attached to N and O were placed at their calculated positions and refined in the isotropic approximation; those attached to heteroatoms (N and O) were located in the difference Fourier maps, and refined with isotropic displacement coefficients. The data were collected at 296(2)K and absorption correction method is none for each case. The crystallographic parameters are given in table 1.

| **Table 1 : Crystal parameters of the compound 1-6** | | | | | | |
| --- | --- | --- | --- | --- | --- | --- |
| Compound No. | **1** | **2** | **3** | **4** | **5** | **6** |
| Formulae | C_24_H_14_N_2_O_3_ | C_18_H_24_N_2_O | C_16_H_12_N_2_O_2_ | C_16_H_12_N_2_O_2_ | C_16_ H_11_ N O_2_ S | C_16_ H_11_ N O_3_ |
| Mol. wt. | 378.37 | 284.39 | 264.28 | 264.28 | 281.32 | 265.26 |
| Crystal system | Monoclinic | Triclinic | Orthorhombic | Orthorhombic | orthorhombic | Triclinic |
| Space group | P2(1)/C | P-1 | Pca2(1) | P2(1)2(1)2(1) | P 2(1) 2(1)2(1) | P-1 |
| *a /Å* | 11.746(3) | 5.465(1) | 24.503(4) | 4.303(8) | 5.3485(5) | 3.8313(5) |
| *b /Å* | 9.579(2) | 11.742(2) | 5.068(8) | 12.249(2) | 7.4424(7) | 12.5135(18) |
| *c /Å* | 17.673(4) | 13.204(2) | 10.750(2) | 23.927(6) | 33.559(3) | 14.2512(19) |
| α/° | 90.00 | 69.478(1) | 90.00 | 90.00 | 90.00 | 112.212(9) |
| β/° | 99.284(2) | 85.113(1) | 90.00 | 90.00 | 90.00 | 93.326(11) |
| γ/° | 90.00 | 85.709(1) | 90.00 | 90.00 | 90.00 | 93.864(11) |
| V/ Å^3^ | 1962.49(8) | 789.7(3) | 1335.0(4) | 1261.0(5) | 1335.9(2) | 628.52(15) |
| Z | 4 | 2 | 4 | 4 | 4 | 2 |
| Density/Mgm^-3^ | 1.281 | 1.196 | 1.315 | 1.392 | 1.399 | 1.402 |
| Abs. Coeff. /mm^-1^ | 0.086 | 0.074 | 0.089 | 0.094 | 0.242 | 0.098 |
| F(000) | 784 | 308 | 552 | 552 | 584 | 276 |
| Total no. of reflections | 10248 | 5807 | 12332 | 10374 | 11033 | 6543 |
| Max. 2θ/° | 24.76 | 28.50 | 29.21 | 28.45 | 28.40 | 28.45 |
| Ranges (h, k, l) | -15 <= h<= 13  -12<= k<=7  -23<= l<= 23 | -7 <= h<= 7  -15<= k<=13  -17<= l<= 13 | -33 <= h<= 33  -6<= k<=6  -12<= l<= 14 | -5 <= h<= 5  -15<= k<=15  -31<= l<= 31 | -7 <= h<= 7  -9<= k<=8  -37<= l<= 44 | -4 <= h<= 5  -16<= k<=16  -18<= l<= 18 |
| Complete to 2θ (%) | 89.5 | 86.9 | 98.5 | 95.65 | 99.3 | 95.6 |
| Refinement method | Full-matrix least-squares  on *F^2^* | Full-matrix least-squares  on *F^2^* | Full-matrix least-squares  on *F^2^* | Full-matrix least-squares on *F^2^* | Full-matrix least-squares on *F^2^* | Full-matrix least-squares on *F^2^* |
| Data/ Restraints/Parameters | 4361/0/272 | 3479/0/200 | 3055/1/186 | 3052/0/185 | 3324/0/189 | 3020/0/189 |
| Goof (*F^2^*) | 0.976 | 0.892 | 0.986 | 0.885 | 1.038 | 1.047 |
| R indices [*I > 2σ(I)*] | 0.0477 | 0.0785 | 0.0538 | 0.0681 | 0.0461 | 0.0669 |
| R indices (all data) | 0.0833 | 0.1760 | 0.1630 | 0.1538 | 0.0961 | 0.1295 |

The crystallographic information of the compounds are deposited to Cambridge Crystallographic Database and has the CCDC numbers 622964, 622965, 628652, 628654, 632764 and 632765.
